# A Multicenter Case Review Program on the impact of ViTAA Regional Aortic Weakness Maps on clinical decision-making in abdominal aortic aneurysm management

**DOI:** 10.1016/j.jvscit.2026.102546

**Published:** 2026-10-07

**Authors:** Alan B. Lumsden, Denis Bilodeau, Case Review Program Working Group, Ponraj Chinnadurai, Marc Schermerhorn, Dennis Gable, John Francis Eidt, Jean Panneton, Sukgu Han, Bernardo Mendes, Federico Ezequiel Parodi, Darren Schneider, Erin Moore, Daniel Clair, Michael C. Stoner, Miranda Witheford, Fahad Shuja, Javairiah Fatima

**Affiliations:** a DeBakey Heart and Vascular Center, Department of Cardiovascular Surgery, Houston Methodist Hospital, Houston, TX; b ViTAA Medical Solutions Inc, Clinical Operations Department, Montreal, QC, Canada

**Keywords:** Abdominal aortic aneurysm, Multiphase gated CTA scans, Static CTA scans, Strain, Intraluminal thrombus, Wall shear stress

## Abstract

**Objective:**

This study, titled the ViTAA Case Review Program, aimed at evaluating the influence of the ViTAA Regional Aortic Weakness (RAW) Maps software on clinical decision-making in the management of abdominal aortic aneurysms (AAA). The purpose was twofold: first, to determine the impact of RAW Maps on projected patient care decisions compared with the standard of care approach, and second, to train investigators in the effective use of ViTAA RAW Maps for AAA assessment. This paper offers a case series focusing on the experience of participating surgeons in using the ViTAA RAW Maps software.

**Methods:**

Fifteen North American vascular surgeons participated and were provided with fully anonymized static and multiphase gated computed tomography angiography (CTA) scans obtained from a previous retrospective study. Five cases were selected from a single site and for each, participating physicians developed hypothetical treatment plans based on two imaging approaches: static CTA scans and multiphase gated CTA scans processed with the ViTAA RAW Maps software. These treatment plans were then compared to determine the impact of the ViTAA approach on clinical decision-making.

**Results:**

The ViTAA approach supported significantly higher surgical intervention rates compared with the standard of care, with vascular surgeons being 69% (*P* < .05) and 88% (*P* < .01) more likely to recommend surgery (either endovascular or open repair) at baseline and follow-up, respectively. The proportion of cases in which the recommendation changed when the ViTAA analysis was provided was 18.9% at baseline (95% confidence interval, 10.0-35.8) and 28.0% at follow-up (95% confidence interval, 19.1-41.0). ViTAA also prompted more endovascular aneurysm repair (72% more at baseline and 57% more at follow-up; *P* = .065 and *P* = .057, respectively), as well as increased fenestrated endovascular aneurysm repair (6.8% vs 2.7% at baseline and 11% vs 7% at follow-up; not statistically significant, *P* = .25 and *P* = .38, respectively). Across the 10 questionnaire items, 73% to 100% of participating physicians responded favorably to questions regarding the functionality and utility of the software.

**Conclusions:**

This study suggests that ViTAA technology may provide additional information for AAA risk stratification by incorporating functional image-derived biomechanical and hemodynamic metrics that offer deeper insight into aortic wall vulnerability. The participating physicians found these additional insights clinically meaningful, with the potential to influence management decisions for the reviewed cases. The usability of the software also suggests feasible integration into future workflows.

**Article Highlights**

• **Type of Research:** “INNOVATIONS in Clinical Care,” a multicenter retrospective review of five anonymized abdominal aortic aneurysm cases by 15 vascular surgeons.
• **Key Findings:** Access to the ViTAA Regional Aortic Weakness Maps led to significantly higher surgical intervention rates compared with the standard of care. ViTAA prompted more endovascular aneurysm repairs and fenestrated endovascular aneurysm repairs at both baseline and follow-up. Physicians found the additional insights into aortic wall vulnerability provided by the ViTAA software to be clinically meaningful and responded favorably to questions regarding its utility.
• **Take Home Message:** The addition of ViTAA Regional Aortic Weakness Maps to conventional imaging changed hypothetical management recommendations in 18.9% of baseline assessments and 28.0% of follow-up assessments. Physicians reported favorable usability; however, prospective studies are needed to establish its clinical benefit.

Abdominal aortic aneurysm (AAA) is defined as a localized enlargement or focal dilation of the abdominal aorta in which the vessel's widest point is at least 1.5 times greater than the normal diameter.^1^ If left untreated, an aneurysm can progressively expand and eventually rupture, which is associated with an elevated mortality rate.^1^, ^2^ AAA affects approximately 0.9% of the global population and, in the United States, a ruptured AAA ranks as the 15^th^ leading cause of death.^3^, ^4^ The currently accepted clinical assessment of rupture risk relies on the aneurysm's maximum diameter and growth rate, with open or endovascular repair being the only available treatments.^5^, ^6^, ^7^ This size-based approach fails to account for localized weakening, the heterogeneity of the aneurysm wall, and individual variability in growth rates.^5^, ^7^, ^8^ Intervention is typically recommended when the diameter is larger than 5.5 cm (5 cm in women) or when there is rapid growth.^7^, ^9^, ^10^, ^11^ However, small aneurysms with a diameter of less than 5 cm can still rupture at rates as high as 12%, while some larger ones may remain stable.^9^, ^12^ All surgeons recognize that although guidelines are based on diameters, there are clearly many more factors which influence timing of aneurysm rupture. The ability to characterize intra- and interpatient aneurysm heterogeneity may provide complementary information for individualized AAA risk assessment. However, whether such information improves risk stratification, changes the appropriateness of treatment decisions, or improves patient outcomes remains uncertain and requires prospective clinical validation.

ViTAA Regional Aortic Weakness (RAW) Maps is an image-analysis software platform that processes multiphase gated computed tomography angiography (CTA) data to generate a three-dimensional visualization of RAW metrics. The software presents a continuous spatial map and a RAW score, reported on a 1 to 10 scale in the version evaluated in this study. Higher values are intended to indicate regions with a greater degree of abnormal wall deformation and/or other incorporated image-derived features relative to lower-valued regions. The RAW score is a proprietary, model-derived output; it is not a direct measurement of histologic wall strength, a molecular biomarker, or a standalone measure of rupture risk.

In this Case Review Program (CRP), RAW Maps were interpreted as adjunctive information to be considered with conventional CTA findings, aneurysm diameter and growth, anatomy, comorbidities, and clinical judgment. No RAW score threshold was prespecified as mandating surveillance, intervention, a particular repair approach, or device selection.

The RAW index integrates three functional components that have been consistently linked to aneurysm progression and rupture risk:

1. Strain (deformability): In vivo principal strain, which is derived directly from multiphase gated CTA images, quantifies local wall deformation without assumptions on material properties. Elevated localized strain has been shown to predict regional weakening and reduced time to rupture.^13^
2. Intraluminal thrombus (ILT) thickness: ILT preferentially develops in regions of disturbed flow and promotes hypoxia and focal inflammation, which is associated with disruption of wall integrity.^6^, ^14^, ^15^, ^16^ Furthermore, ILT thickness is positively correlated with markers of tissue degeneration.^7^
3. Wall shear stress (WSS): Low time-averaged WSS (TAWSS) contributes to ILT deposition and unfavorable wall remodeling.^15^ Regions of low TAWSS with concomitant high strain and thick ILT represent areas at particularly high risk.^9^, ^17^

The RAW index identifies regions where low TAWSS, thick ILT, and elevated strain converge, capturing the heterogeneous weakening processes that underlie aneurysm growth and rupture.^5^ More recently, this compound measure was shown to accurately identify faster than average AAA growth, underscoring its potential as a clinically relevant biomarker of disease progression.^18^ The index also highlights focal areas of structural vulnerability that may not correspond to the site of maximum aortic diameter.

The present Case Review Program was not designed to validate RAW Maps as a risk-stratification tool and did not evaluate patient outcomes. Rather, it examined whether access to RAW Maps was associated with differences in vascular surgeons' hypothetical care plans compared with review of static CTA images alone.

## Methods

### Materials provided

Fifteen geographically dispersed North American vascular surgeons participated in this retrospective review of five anonymized AAA cases and were provided with access to the ViTAA RAW Maps WebApp and a DACIMA account for data capture, as well as the User Manual, study guides, training videos, and a feedback form (Supplementary Documents 1 and 2, online only). The physicians completed their assessments using an electronic case report form (CRF) via a web-based data capture system managed by the DACIMA Clinical Suite, according to Food and Drug Administration 21 Code of Federal Regulations part 11 requirements, Health Insurance Portability and Accountability Act specifications, and International Conference on Harmonization standards (Supplementary Document 1, online only).^19^ The DICOMs used for the five patients were fully anonymized static and multiphase-gated CTA scans obtained from the study *Dynamic Scanning for the Prediction of Aortic Aneurysm Growth: A Retrospective Review of CT Images* (Ethics ID: REB21-0043; Protocol No. CIP-ViTAA-002) conducted at the Peter Lougheed Centre in Alberta, Canada. These cases, acquired as part of clinical monitoring, included at least two serial abdominal-pelvic multiphase gated CTA scans, at both baseline and follow-up. All CTA scans were fully deidentified before distribution, with each image file assigned a Global Unique Identifier, which was shared with the vascular surgeons.

### Study population

The retrospective study focused on patients who had been monitored through multiphase gated CTA imaging and who had at least two multiphase CTA scans. Patients over the age of 18 years with a diagnosis of AAA disease and multiphase-gated CTA scans from January 1, 2016, to December 2022 were included. Exclusion criteria included a follow-up of aneurysm disease without multiphase-gated scanning, the absence of a follow-up scan completed at least 8 months following the initial scan, or patients with follow-up CTA scans done more than 24 months postbaseline.

### Study design

Baseline and follow-up images were assessed using each surgeon's preferred imaging software to measure aneurysm characteristics, including maximum diameter and neck length. For comparative practice analysis surgeons had access to the ViTAA RAW Maps software along with the reports and the RAW Maps generated for each patient.

Each surgeon independently completed assessments in an electronic CRF platform (DACIMA) and developed theoretical care plans using two approaches: (1) standard of care (SOC) review of static CTA images and (2) ViTAA RAW Maps analysis of multiphase-gated CTA scans. Plans included recommendations on timing of intervention, procedure type, and device selection, which were subsequently compared across participants. The study design workflow is summarized in Fig 1.

**Fig 1:**
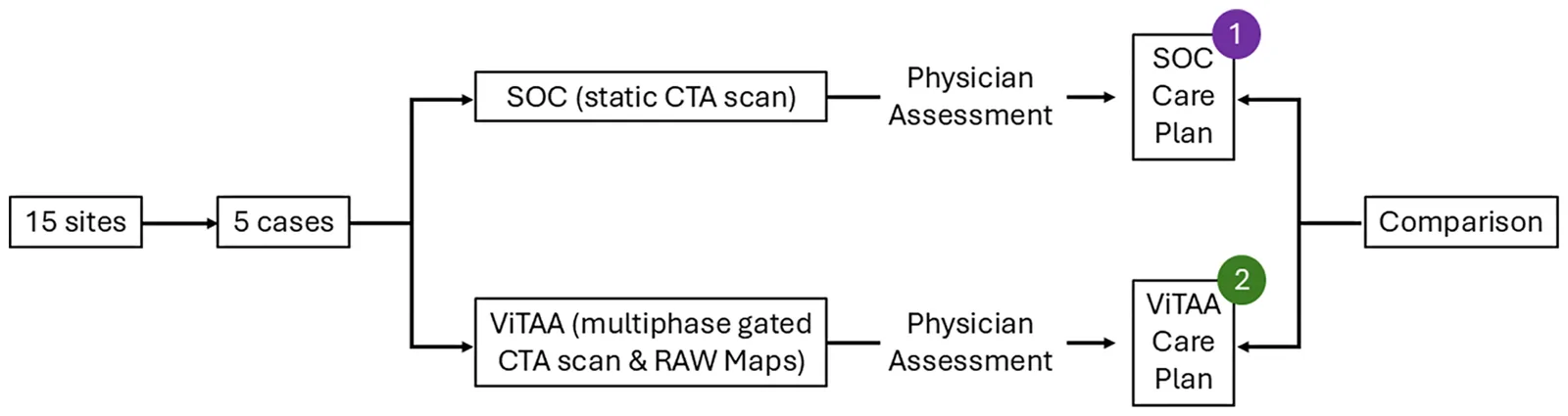
The study design workflow. Fifteen vascular surgeons, geographically dispersed across multiple centers, retrospectively reviewed five anonymized abdominal aortic aneurysms (AAA) cases obtained from the study Dynamic Scanning for the Prediction of Aortic Aneurysm Growth: A Retrospective Review of Computed Tomography (CT) Images (Ethics ID: REB21-0043; Protocol No. CIP-ViTAA-002) conducted at the Peter Lougheed Centre in Alberta, Canada. For every patient, each participating clinician developed hypothetical treatment plans based on (1) static computed tomography angiography (*CTA*) scans, representing the standard of care (*SOC*), and (2) multiphase gated CTA scans processed with the ViTAA Regional Aortic Weakness (*RAW*) Maps software, representing the ViTAA approach. Finally, the SOC and ViTAA care plans were compared to determine the impact of RAW Maps on the projected patient care decisions.

The two approaches (SOC and ViTAA) were applied sequentially by the vascular surgeons rather than concurrently. For each case and at each time point, investigators first performed the SOC assessment using their preferred imaging software and recorded the resulting measurements and projected care plan, including timing of assessment, type of intervention, type of procedure, and timing of follow-up. The ViTAA RAW Maps reports were then reviewed. The previously recorded SOC plan was transcribed into the ViTAA section of the CRF, and investigators indicated which modifications, if any, they would make based on the ViTAA data. Each SOC plan was therefore established and documented before any exposure to ViTAA output.

The care plans were hypothetical and were not communicated to treating clinicians for implementation. No patient was randomized, no treatment was assigned on the basis of RAW Maps, and no clinical outcomes, treatment appropriateness, surveillance outcomes, rupture events, or adverse events were evaluated as study end points.

The physicians were also trained on the effective use of the ViTAA RAW Maps software in AAA assessment during real-time remote structured training sessions. At the end of the study, the physicians were asked to respond to a feedback form (a structured questionnaire designed to gather user feedback on both the usability and the overall confidence in using the software). Finally, a live Clinical Outcome Reveal session was conducted to discuss the therapeutic decisions across the five cases.

### Statistical methods

Hypothetical care plans generated with and without ViTAA information were compared at baseline and follow-up. Relative risks of recommending a procedure were estimated using a generalized linear model with a binomial distribution and log link.^20^, ^21^ Because each case was assessed by multiple clinicians across centers, observations were clustered, and robust variance estimates were obtained using the Huber-White sandwich estimator to account for within-cluster correlation.^22^, ^23^, ^24^, ^25^ Relative risks and 95% confidence intervals (CIs) were expressed as a percentage of increases in recommended procedures, and descriptive analyses summarized the types of procedures and medication classes. For paired binary comparisons of specific procedure subtypes [eg, fenestrated endovascular aneurysm repair (FEVAR)], McNemar exact test was used given the paired nature of the SOC and ViTAA assessments.

## Results

### Clinical profiles and outcomes

The clinical profiles and outcomes of the five anonymized, retrospective cases of AAA are summarized further.

#### Case CRP-001

A 70-year-old man had chronic obstructive pulmonary disease (COPD), coronary artery disease, and a small 4.6 cm AAA (Fig 2). There were areas of high initial RAW located throughout the aorta, especially close to the neck/sac junction superiorly. Although his initial RAW scores prompted a discussion with the patient regarding possible early repair, his comorbidities were prohibitive, and the patient chose to defer treatment. However, due to rapid growth, he had semiurgent endovascular aneurysm repair (EVAR) for a 5.8 cm AAA 3 years after his initial scan. His postoperative images demonstrated no endoleaks, and he survived for 3 years before succumbing to metastatic lung cancer in 2023.

**Fig 2:**
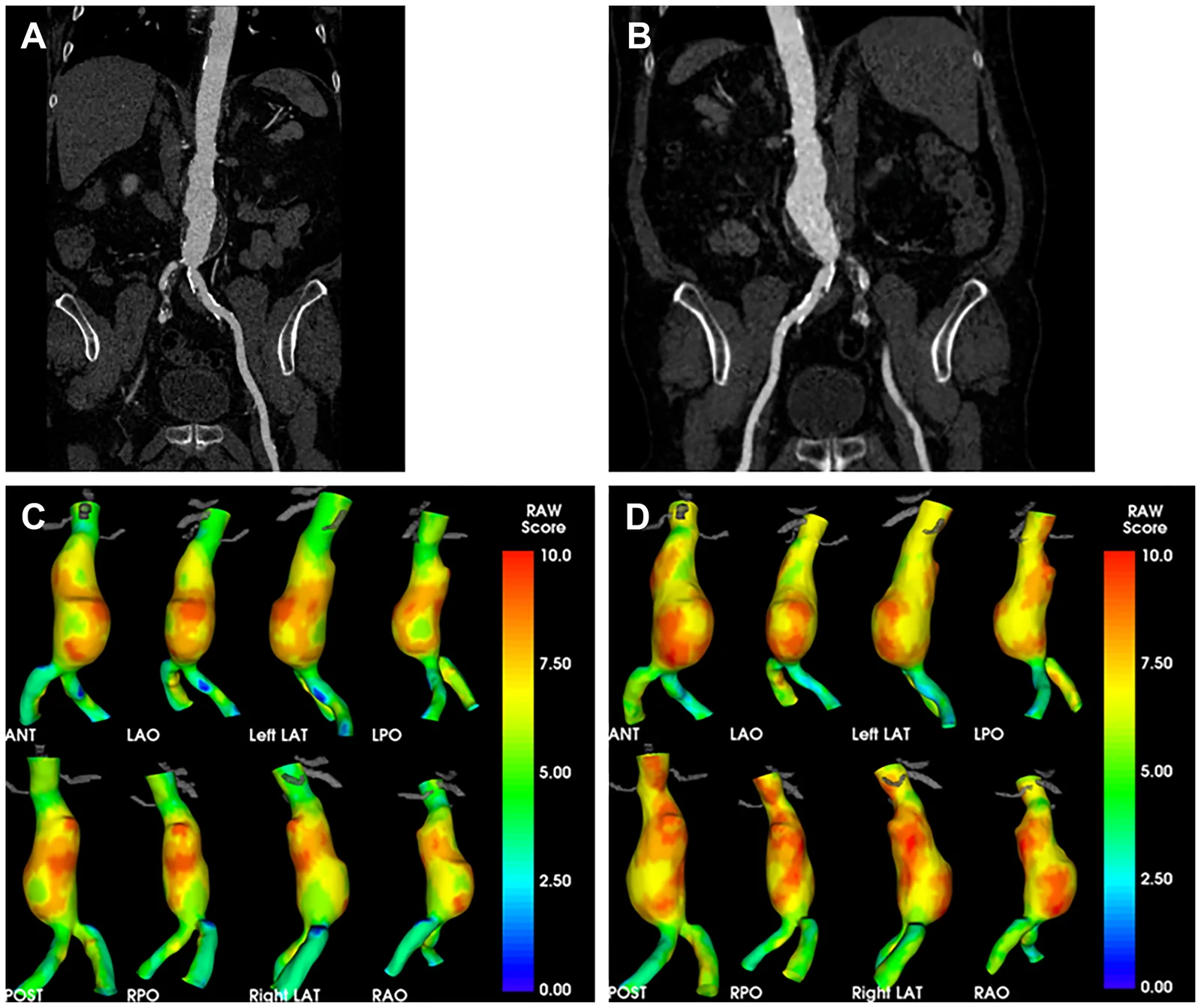
Computed tomography (CT) scans and continuous Regional Aortic Weakness (*RAW*) Maps of patient CRP-001. **A** and **B,** CT MPR demonstrating a small, benign-appearing abdominal aortic aneurysm (AAA). **C,** The RAW Maps at baseline show high RAW scores after ViTAA analysis. **D,** Follow-up Maps show progression of RAW with growth, which led to semiurgent endovascular aneurysm repair (EVAR). A higher RAW score indicates an aortic area that is weaker than areas with lower scores. The continuous representation shows a RAW score continuously over the aorta. *LAO*, Left anterior oblique; *LAT*, lateral; *LPO*, left posterior oblique; *RPO*, right posterior oblique; *RAO*, right anterior oblique.

#### Case CRP-002

A 79-year-old man had COPD and was not on supplemental oxygen. The patient's initial scan demonstrated multiple areas of increased RAW, and his aneurysm grew from 4.7 to 5.2 cm over 3 years (Fig 3). The patient was offered a fenestrated repair due to his short, reverse conical neck, but the patient lived remotely and did not want to come back for serial follow-up. The patient had open juxtarenal repair in 2020 with pledgeted sutures due to poor tissue quality. There were no postoperative complications; however, the patient died in 2024 due to severe pulmonary failure and COPD exacerbation.

**Fig 3:**
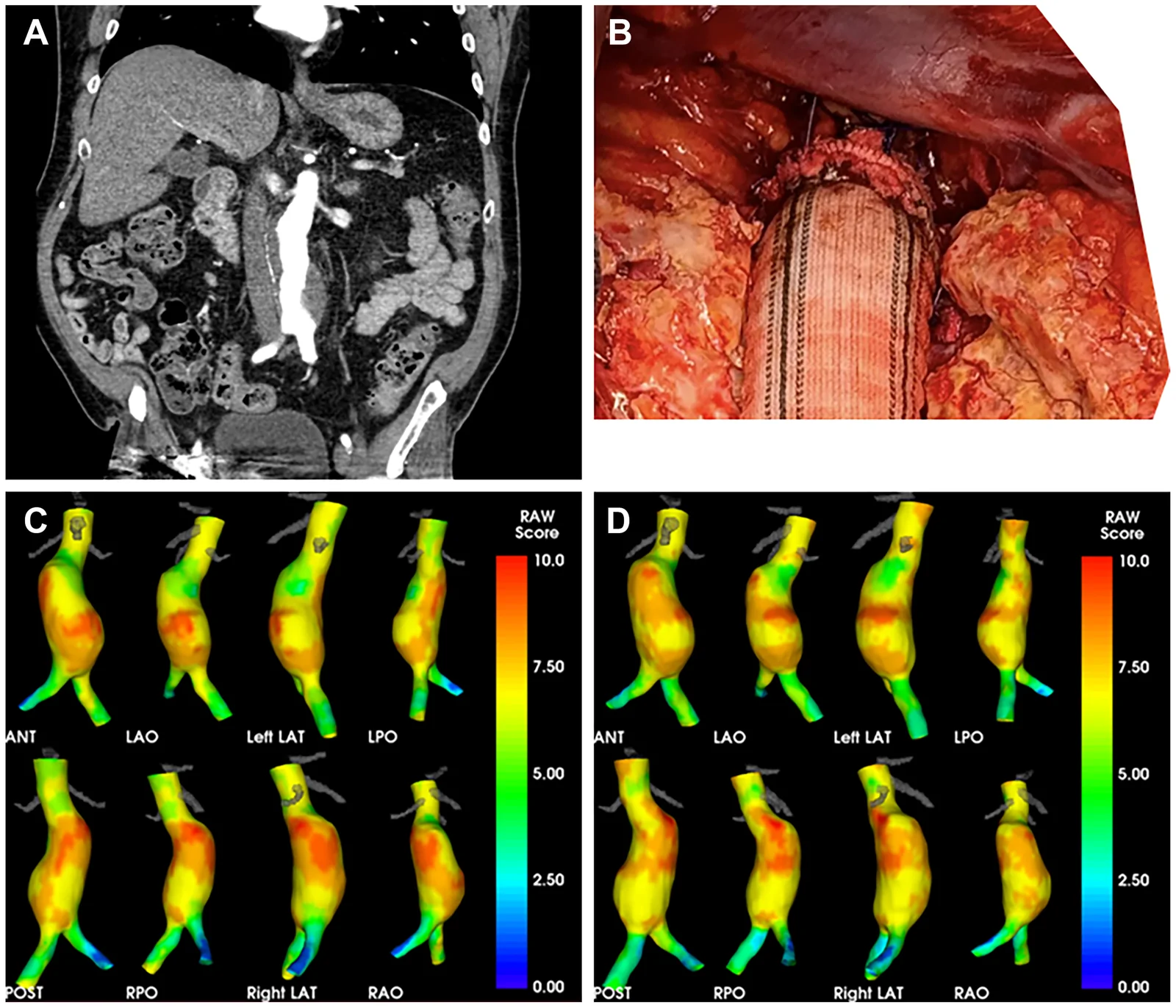
Computed tomography (CT) scan and continuous Regional Aortic Weakness (RAW) Maps of patient CRP-002. **A,** CT scan of the patient showing a short, reverse conical neck. **B,** This required pledgeted anastomosis due to the poor tissue quality identified by ViTAA analysis. **C,** Baseline RAW Maps. **D,** Follow-up Maps show a slowly enlarging abdominal aortic aneurysm (AAA) that exceeds 5.0 cm and high RAW scores within multiple areas in the infrarenal neck. A higher RAW score indicates an aortic area which is weaker than those areas with a lower score. The continuous representation shows a RAW score continuously over the aorta. *LAO*, Left anterior oblique; *LAT*, lateral; *LPO*, left posterior oblique; *RPO*, right posterior oblique; *RAO*, right anterior oblique.

#### Case CRP-003

A 64-year-old man lived remotely and away from medical care. He had a small infrarenal AAA measuring 5.2 cm, untreated severe hypertension, and elevated RAW scores (Fig 4). After a long discussion with the patient and serial ViTAA imaging that demonstrated good quality (low RAW) at the landing zones, along with improved RAW scores after initiating appropriate antihypertensive therapy, early EVAR was performed below intervention threshold in 2019. His sac has continued to regress, with no endoleaks to date.

**Fig 4:**
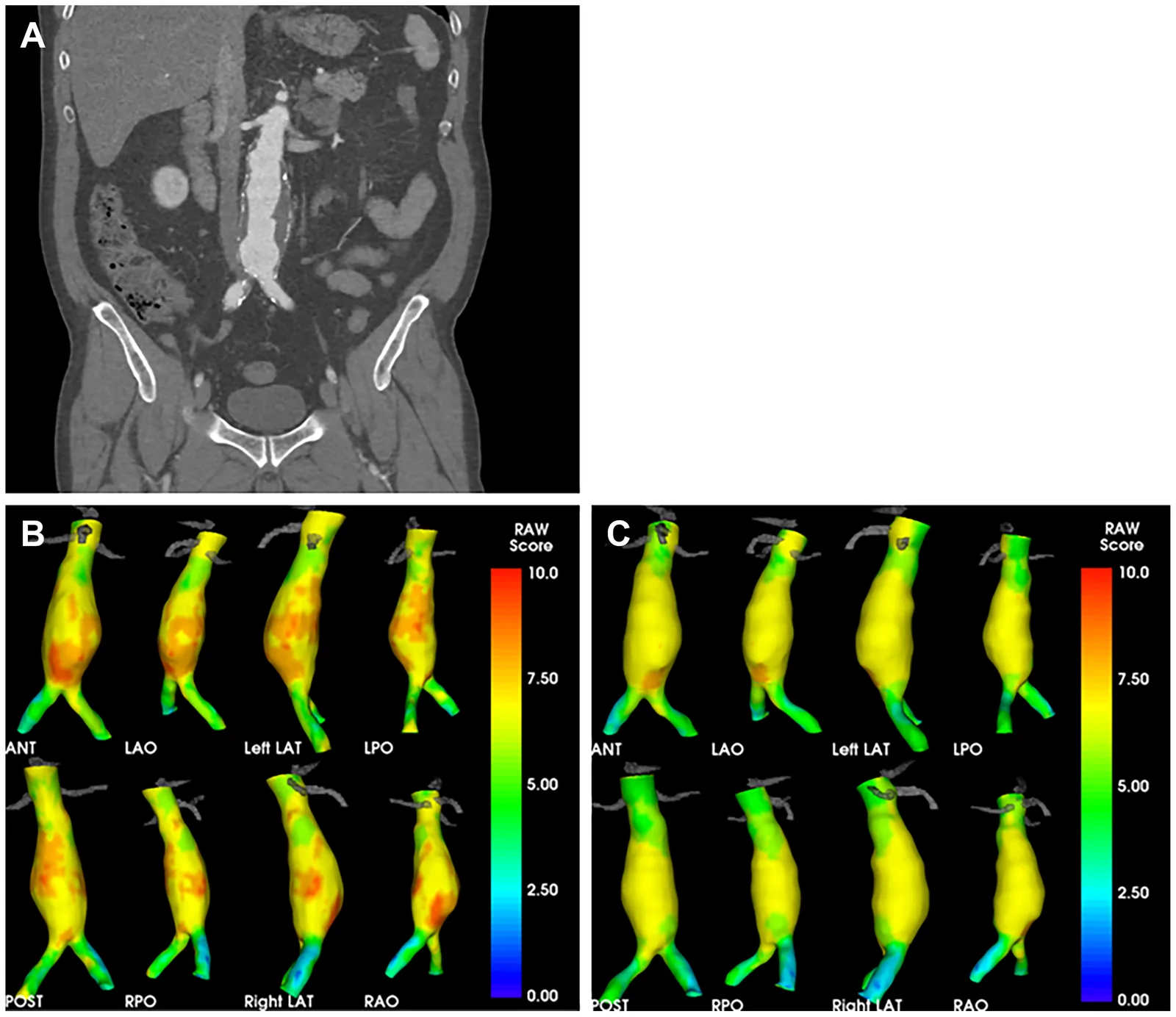
Computed tomography (CT) scan and continuous Regional Aortic Weakness (RAW) Maps of patient CRP-003. **A,** CT scan of the patient with a 5.2 cm abdominal aortic aneurysm (AAA) and severe uncontrolled hypertension. **B,** Baseline RAW Maps showing elevated RAW scores in multiple regions. **C,** Follow-up RAW Maps show improved RAW due to reduced strain related to antihypertensive medications. A higher RAW score indicates an aortic area which is weaker than those areas with a lower score. The continuous representation shows a RAW score continuously over the aorta. *LAO*, Left anterior oblique; *LAT*, lateral; *LPO*, left posterior oblique; *RPO*, right posterior oblique; *RAO*, right anterior oblique.

#### Case CRP-004

An 84-year-old man had an extremely small body habitus (the patient weighed 40 kg). He had slow serial dilation of the aorta to 5.2 cm and provided consent for surgery based on saccular configuration, the disproportionately large AAA relative to his small body size, and RAW Maps findings demonstrating excellent tissue integrity at all landing zones except the right iliac artery, which was coiled and covered (Fig 5). To date, the patient's follow-up CTA scans demonstrate sac regression despite a very small type 2 lumbar endoleak (arrow).

**Fig 5:**
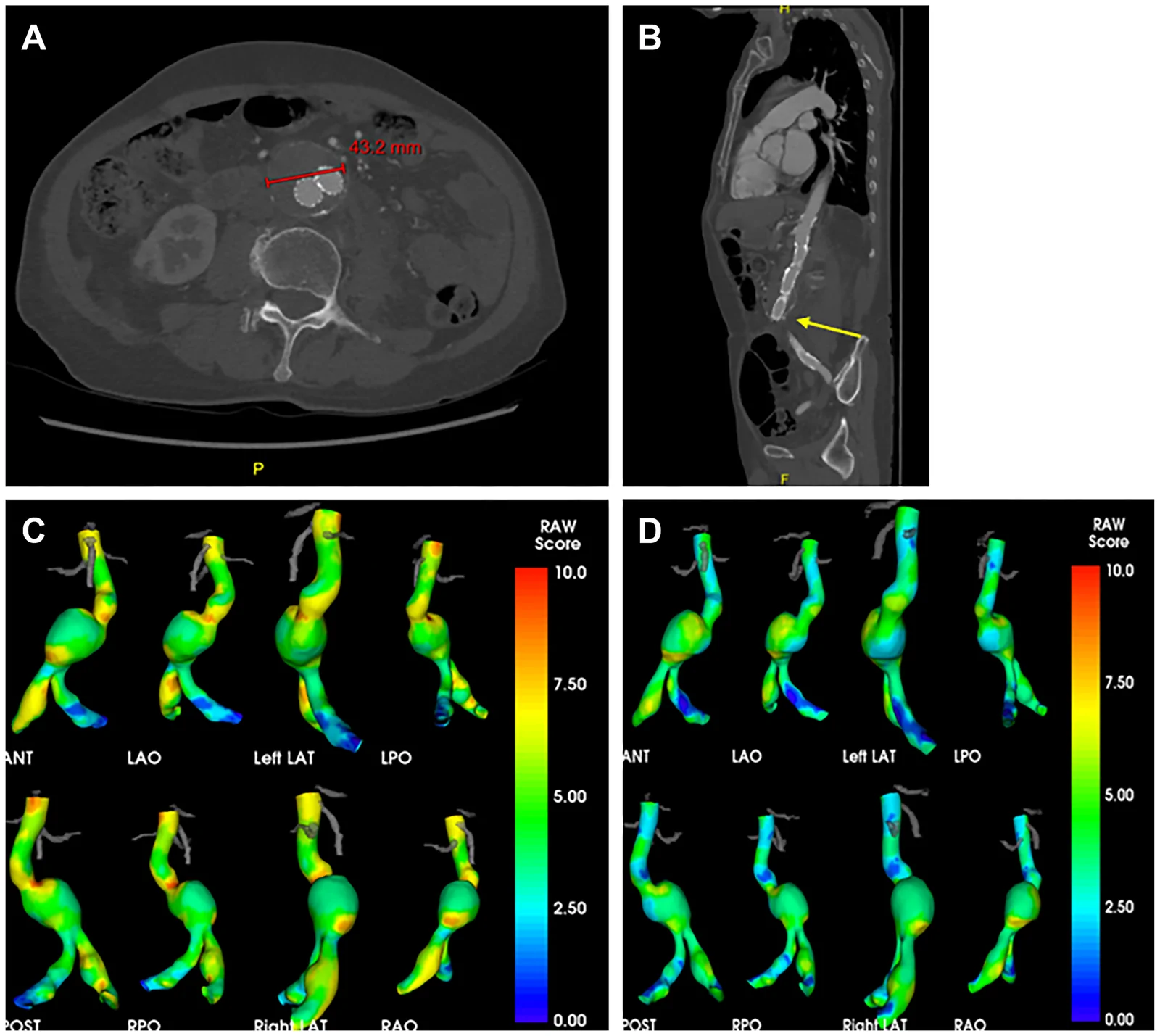
Computed tomography (CT) scans and continuous Regional Aortic Weakness (RAW) Maps of patient CRP-004. **A** and **B,** CT scans of the patient showing a small abdominal aortic aneurysm (AAA). To date, follow-up CT scans demonstrate regression despite the presence of a very small type 2 lumbar endoleak (*arrow*). **C,** Baseline RAW Maps show high RAW at the distal right iliac landing zone. **D,** Follow-up RAW Maps. A higher RAW score indicates an aortic area which is weaker than those areas with a lower score. The continuous representation shows a RAW score continuously over the aorta. *LAO*, Left anterior oblique; *LAT*, lateral; *LPO*, left posterior oblique; *RPO*, right posterior oblique; *RAO*, right anterior oblique.

#### Case CRP-005

A 73-year-old woman had a small AAA that had enlarged from 4.1 to 4.8 cm over 4 years. She had also developed significant thrombus at the aortic infrarenal neck along with a penetrating atherosclerotic ulcer immediately inferior to the right renal artery, which was associated with marked elevation of her RAW scores in this area, which precluded safe endovascular repair (Fig 6). The patient received an open bifurcated repair with a supraceliac aortic clamp. At the time of her open repair, her tissue integrity was compromised, which required extra sutures to buttress. She then developed massive postoperative bleeding due to anastomotic tears at the neck, multiple systems organ failure and died of sepsis 30 days after the repair.

**Fig 6:**
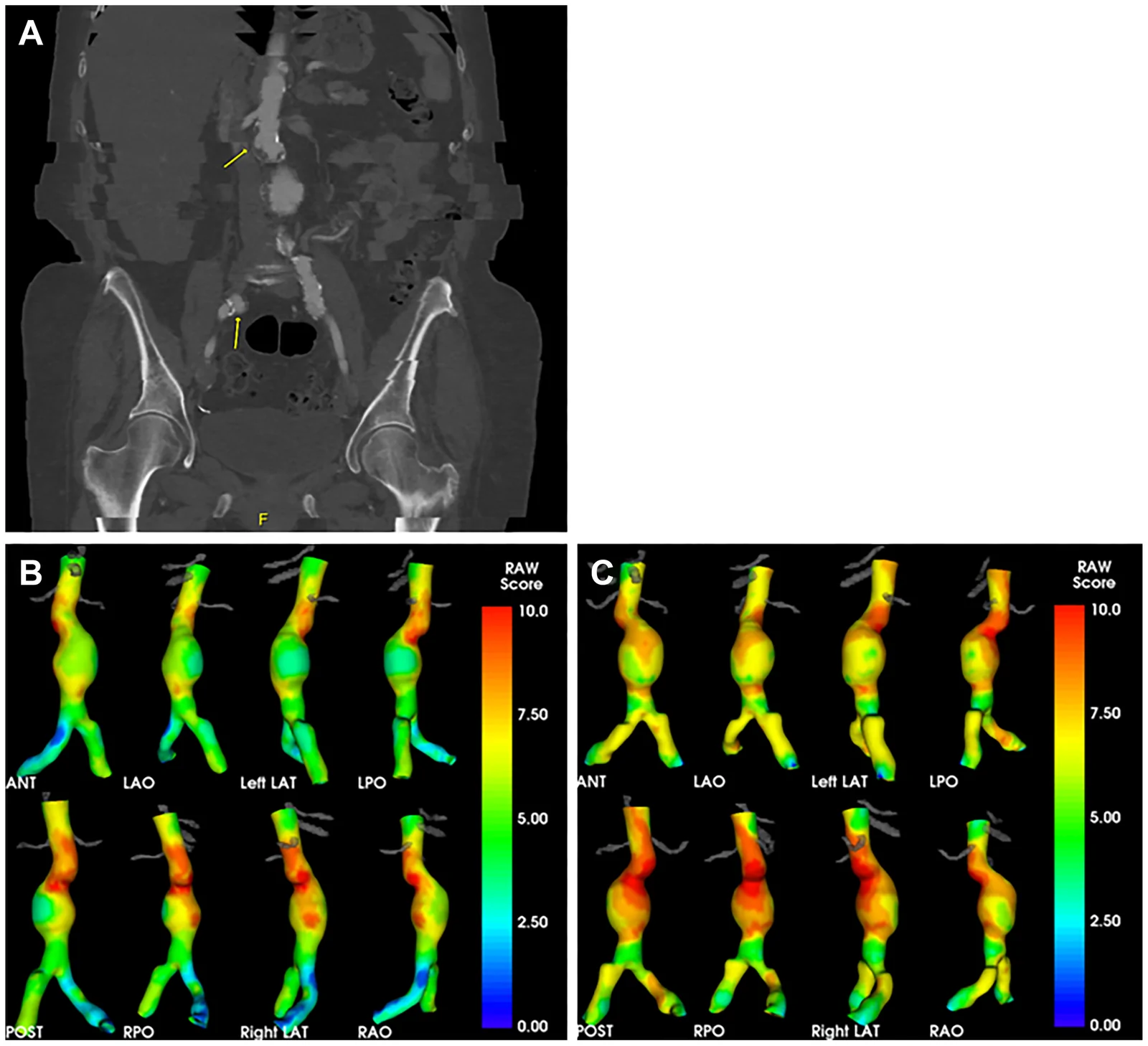
Computed tomography (CT) scan and continuous Regional Aortic Weakness (RAW) Maps of patient CRP-005. **A,** computed tomography (CT) scan of the patient with significant thrombus at the aortic infrarenal neck and a penetrating atherosclerotic ulcer (PAU) immediately inferior to the right renal artery. Elevated RAW scores at the thrombotic neck and right iliac PAU (*arrows*) precluded safe endovascular aneurysm repair (EVAR). **B,** Baseline RAW Maps. **C,** Follow-up RAW Maps showing marked elevation of the patient's RAW scores immediately inferior to the right renal artery, as well as at the thrombotic neck. A higher RAW score indicates an aortic area which is weaker than those areas with a lower score. The continuous representation shows a RAW score continuously over the aorta. *LAO*, Left anterior oblique; *LAT*, lateral; *LPO*, left posterior oblique; *RPO*, right posterior oblique; *RAO*, right anterior oblique.

### Impact of ViTAA on AAA treatment decisions

#### Likelihood of procedures

In the evaluation of AAAs, the use of ViTAA for the cohort of patients examined was associated with a higher frequency of surgical recommendations (endovascular or open repair) compared with the SOC (Table). At baseline, the probability of recommending intervention was 69% greater with ViTAA (*P* < .05). At follow-up, the probability increased to 88% (*P* < .01) when decisions were based on ViTAA rather than SOC imaging alone. At baseline, the proportion of cases in which the recommendation changed when the ViTAA analysis was provided was 18.9% (95% CI, 10.0-35.8). At follow-up, this proportion was 28.0% (95% CI, 19.1-41.0).

**Table:** Proportion of patients requiring a procedure: ViTAA vs standard of care (*SOC*)

Time point	SOC (n/N)	SOC (%)	ViTAA (n/N)	ViTAA (%)	RR (95% CI)	% Increase (95% CI)	*P* value
Baseline	12/75	16.0	20/74	27.0	1.69 (1.09-2.63)	69% (9%-163%)	.020
Follow-up	17/75	22.7	32/75	42.7	1.88 (1.30-2.72)	88% (30%-172%)	.001

*CI*, Confidence interval; *RR*, relative risk. One baseline ViTAA-based recommendation was not recorded; this assessment was excluded from the baseline ViTAA proportion and paired comparisons (n = 74).

#### Procedure type

When evaluating physician recommendations for the type of intervention, ViTAA led to a notable shift in clinical decision-making. At baseline, ViTAA usage resulted in a 72% increase in EVAR procedures compared with the SOC (McNemar exact test, *P* = .065), a difference that trended toward but did not reach statistical significance. This pattern was consistent at follow-up, where EVAR was selected 57% more often with ViTAA, again approaching but not reaching statistical significance (*P* = .057).

#### Types of endovascular repair

At baseline, the use of ViTAA changed the recommended type of intervention in all five evaluated cases. Recommendations for FEVAR were numerically more frequent with ViTAA (6.8%) than the SOC (2.7%), although this difference was not statistically significant (McNemar exact test, *P* = .25). At follow-up, the ViTAA platform resulted in a change in procedure type in all five reviewed cases, and FEVAR was recommended in 11% of ViTAA-based assessments vs 7% of SOC-based assessments; this difference was also not statistically significant (*P* = .38). Given the small number of discordant assessments, these FEVAR findings should be interpreted as exploratory.

### Overall physician feedback

#### Software usability feedback

Physicians found ViTAA RAW Maps to be a usable and valuable tool for patient monitoring, highlighting its intuitive interface and features such as side-by-side comparison at baseline and follow-up maps and integrated measurement tools. Some challenges were noted, particularly with infrequent use, complex aortic measurements, and interpreting the novel RAW scores. Physicians suggested enhancements to PDF report usability, integration with visualization tools, and additional functionalities such as stent graft or full case planning. Participating physicians identified potential future applications in AAA surveillance; however, the present study did not evaluate predictive performance or clinical outcomes. Prospective studies are required to determine whether RAW Maps has clinical utility in surveillance. Eighty percent of participating physicians answered “yes” to the item asking whether the software was easy to navigate. Across all 10 directive questionnaire items, the proportion of physicians answering “yes” ranged from 73% to 100% (Supplementary Table I, online only). This range reflects item-level responses and is not an overall usability score.

#### ViTAA metrics impacting decisions

Fig 7 summarizes the ViTAA metrics most likely to impact the vascular surgeons' decision-making process, both at baseline and follow-up. Interestingly, the aortic diameter metric was rated very low (5.3%) in terms of decision-making impact compared with the RAW metrics (RAW score value, location, and distribution).

**Fig 7:**
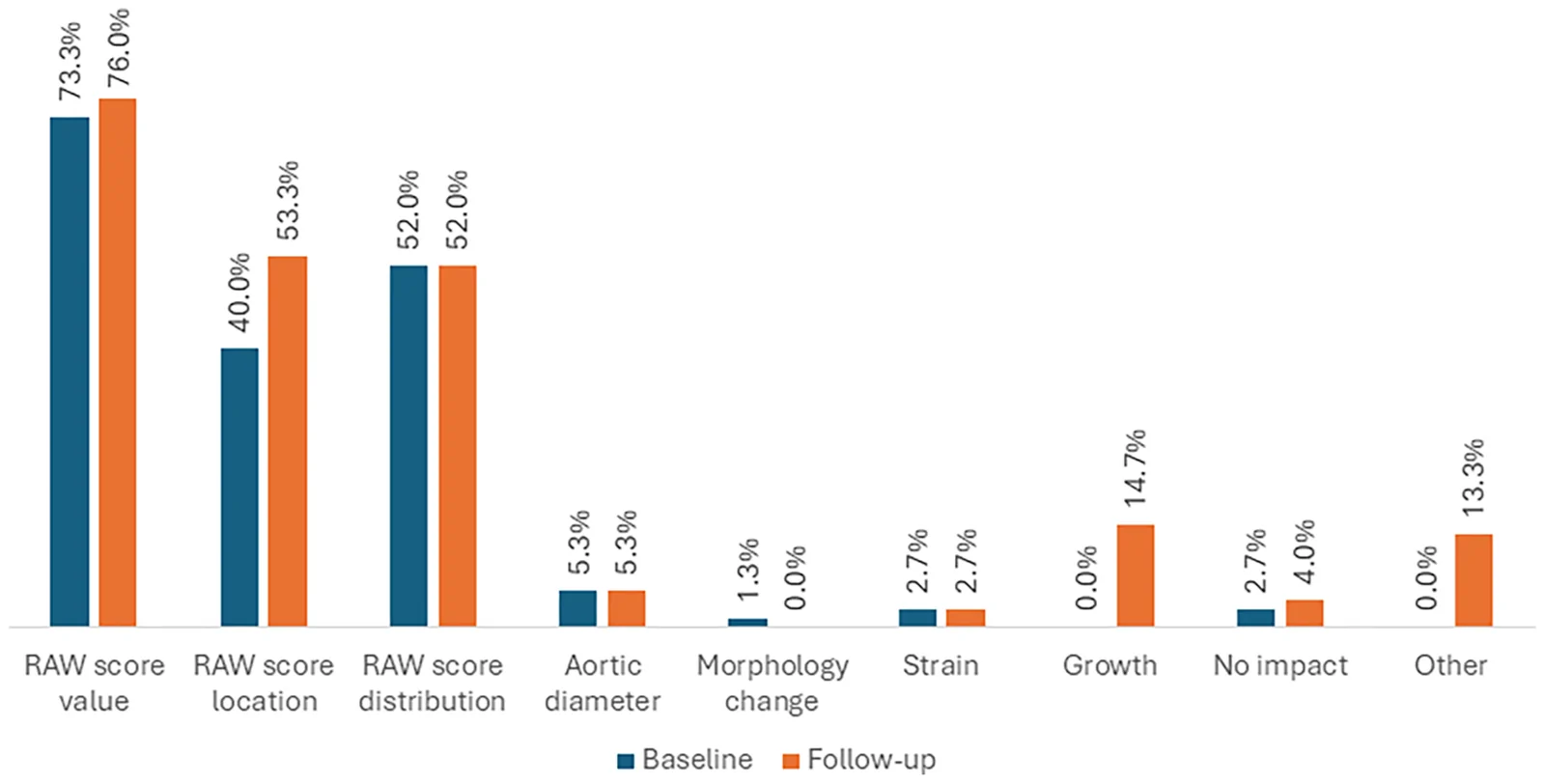
ViTAA metrics impacting the decision-making process. Baseline values are shown in *blue*, whereas follow-up values are shown in *orange*.

## Discussion

### Study limitations

This study provided important insights into the role of advanced imaging in the management of AAAs. However, there are important limitations that should be considered when interpreting its findings. First, only five retrospective AAA cases were reviewed, limiting statistical power, precision, and generalizability. Second, the number of participating vascular surgeons was limited, and the surgeon-level sample restricts inference about broader clinician practice patterns. Third, the cases were selected from a pre-existing database and may not represent the spectrum of AAA anatomy, disease stage, sex, race/ethnicity, comorbidity, or imaging quality encountered in routine practice. Fourth, the care plans were hypothetical, and no patient outcomes, treatment appropriateness, or clinical utility end points were evaluated. Fifth, the review workflow may have introduced review-order or information-presentation bias. Finally, the directive questionnaire used binary yes/no responses, which limited the ability to capture gradations in usability, interpretability, perceived clinical relevance, and confidence. Future studies should use prespecified Likert-type response scales (eg, 1 = “not at all”; 5 = “very”) and, where feasible, validated usability instruments. Although the theoretical care plans developed in the study are not yet validated through clinical interventions or patient follow-up, they serve as a crucial first step in understanding how tools such as the ViTAA RAW Maps may optimize treatment strategies by refining intervention timing, choice of procedure, and device selection.

### Impact of ViTAA RAW maps on clinical decision-making

The main finding of this study was that access to ViTAA RAW Maps was associated with differences in projected care plans compared with reliance on static CTA images alone. These findings describe how participating surgeons used additional image-derived information in a hypothetical review setting; they do not establish improved AAA risk stratification, treatment appropriateness, or patient outcomes. Depending on the wall characteristics revealed by the ViTAA Maps, some clinicians chose to adjust their hypothetical intervention timing, select alternative procedures, or recommend more intensive surveillance. For example, when the ViTAA Maps showed focal areas of high strain or thick ILT that were not apparent by the maximum diameter criteria alone, physicians favored earlier intervention. In the absence of high-risk features, the software enabled a conservative management plan.

To delve deeper into the decision-making processes, participants were asked to further review one of the five cases. Upon completion of the study and subsequent analysis, physicians who were asked to review the same patient were grouped into subteams within which they collaboratively reached a consensus regarding each case (Supplementary Table II, online only).

### Physician consensus: CRP-001

After the data was reviewed using the two strategies proposed in the study (SOC and the ViTAA software), there was an overall consensus at baseline (for both the SOC and ViTAA analysis) that no surgery or intervention was indicated, and follow-up at 6 to 12 months was acceptable with ongoing medical management. At the 12-month follow-up window, the consensus by SOC was still for medical management, but given the growth of the aneurysm, the follow-up window was shortened to 6 months. When ViTAA analysis was used for evaluation at the follow-up window, the consensus for treatment was continued medical management with a shortened follow-up window of 6 months. There was a unanimous heightened level of concern for a small amount of growth and worsening RAW image findings. The reviewers noted that both the RAW score at specific locations and the overall distribution changes across the entire aorta were critical to the decision-making process. This finding goes directly to the application that an increase in overall RAW score, not just the finite location of any change, may be important. In fact, it has been shown that patients with an overall change may have an increased risk for volumetric growth as well as a possible risk for rupture.

### Physician consensus: CRP-002

We are more likely to intervene earlier with the RAW information as we are incorporating it into our decision making rather than relying on diameter and growth rate alone.

### Physician consensus: CRP-003

Based on imaging review, both physicians agreed that continued surveillance with duplex ultrasound at 6-month intervals was appropriate until interval growth warranted further evaluation with CTA. Upon review of the RAW Maps, we identified several regions of increased “thermal” activity, suggesting multiple diffuse areas of weakness. The patient was also found to have uncontrolled hypertension. Interestingly, once his blood pressure was optimized with antihypertensive therapy, subsequent RAW Map analyses demonstrated a marked reduction in “heat” intensity, whereas aneurysm size showed only a slight increase from 5.0 to 5.2 cm.

Although the aneurysm diameter remained borderline for intervention by current standard guidelines, the decrease (assumed improvement) in the RAW Map areas of intensity raises some uncertainty regarding timing and approach to repair. In this test case, the operating surgeon ultimately elected to proceed with endovascular repair (EVAR). The RAW Maps proved particularly useful in defining the optimal proximal landing zone. Despite a relatively short neck, the region demonstrated low RAW Map readings, providing reassurance regarding its suitability as a durable sealing zone. Conversely, had the proximal neck demonstrated high “heat” activity, the team would have likely opted for a higher landing zone, favoring a fenestrated approach (FEVAR).

Overall, the RAW Maps provided valuable confidence in selecting EVAR as a durable treatment strategy. Postoperative outcomes have been consistent with preoperative ViTAA imaging predictions, including aneurysm sac regression—a known marker of EVAR success. This case highlights the utility of ViTAA imaging not only in refining the timing of intervention but also in guiding selection of the most appropriate and durable repair modality. The system leaves opportunity for further understanding and discussion about rupture predictability as the adjustment to the patient's hypertension resulted in an apparent decrease in wall stress imaging.

### Physician consensus: CRP-004

The measured maximum AAA diameter ranged from 39 to 51 mm among the participating physicians using three different 3D MPR software. Medical management was consistent, including cholesterol lowering drugs and beta blockers. Two physicians elected to use anticoagulant/anti aggregation agents, and one elected to add angiotensin II receptor blockers. ViTAA did not change the medical management regimen. Based on the SOC measurements, two out of three physicians elected for continued medical management with 6 months as the appropriate interval for surveillance, and one elected for open repair. ViTAA RAW score led to the change in management in one physician from open repair to continued medical management.

### Physician consensus: CRP-005

The RAW Map as well as the CT scan with the new penetrating atherosclerotic ulcer just below the right renal artery clearly showed that an infrarenal device would very likely not be successful. Based on this information, the primary surgeon decided to place a supraceliac clamp during the open repair. The tissue in this high RAW segment was very frail and not holding stitches, resulting in unfavorable outcome. This information underscores the potential risk of rupture, the possible failure of endovascular repair, the required extent of endovascular intervention, and provides guidance on optimal sites for suturing an open graft.

The success of endovascular aortic aneurysm repair hinges on choosing an appropriate site for achieving proximal and distal seal. Presence of thrombus or calcification is considered unfavorable for achieving adequate graft apposition. Furthermore, tissue frailty raises the likelihood of anastomotic challenges and complications. Tissue frailty is difficult to predict on our existing diagnostic modalities. The ViTAA RAW score considers flow disturbances, thrombus burden, and wall deformation, and has been validated to identify areas of aortic wall weakness. This has profound implications for operative planning, both endovascular and open. The case highlights the value of using the ViTAA RAW score in choosing sites for aortic clamping and anastomosis.

## Conclusions

ViTAA assessments resulted in higher intervention rates compared to SOC, with surgeons 69% more likely to intervene at baseline and 88% more at follow-up in the cohort examined. ViTAA prompted more EVARs (72% more at baseline, 57% more at follow-up; *P* = .065 and *P* = .057, respectively, approaching but not reaching statistical significance) and a numerically higher, but not statistically significant, proportion of FEVARs (6.8% vs 2.7% at baseline; 11% vs 7% at follow-up; *P* = .25 and *P* = .38, respectively).

In this small retrospective case-review program, access to ViTAA RAW Maps was associated with differences in surgeons' hypothetical AAA care plans and was perceived as usable by participating physicians. These findings are hypothesis-generating and do not establish improved AAA risk stratification, clinical benefit, treatment appropriateness, or improved patient outcomes. Prospective studies with prespecified clinical end points and patient follow-up are required.

This preliminary retrospective analysis demonstrated that the ViTAA graphical depiction of regions of aortic wall weakness may significantly impact AAA care planning by integrating image-derived biomechanical and hemodynamic metrics. The ViTAA Maps also provide deeper insight into aortic wall vulnerability than traditional static imaging alone. In conclusion, this study offers promising evidence that image-derived metrics and digital decision-support tools may provide additional information for AAA assessment. In this study, access to RAW Maps was associated with differences in hypothetical care plans; whether the software improves the accuracy or precision of AAA risk assessment, surveillance, intervention timing, or device selection remains unknown and requires prospective clinical evaluation.

## Author Contributions

Conception and design: AL, DB

Analysis and interpretation: AL, DB

Data collection: AL

Writing the article: AL, DB

Critical revision of the article: AL, DB

Final approval of the article: AL, DB

Statistical analysis: Not applicable

Obtained funding: DB

Overall responsibility: DB

## Ethical considerations

The Retrospective Study (CIP-ViTAA-002), from which the five AAA cases were selected for the Case Review Program, was approved by the Conjoint Health Research Ethics Board at the University of Calgary (Ethics ID: REB21-0043). WCG granted an exemption for the Case Review Program because of the absence of new data collection. Additional Institutional Review Board (IRB) approvals were obtained at some participating institutions, including Houston Methodist Medical Center and Beth Israel Deaconess Medical Center. Informed consent was not required, and all images were anonymized using study subject IDs and sponsor-provided globally unique identifiers (GUIDs).

## Patient consent

Patient informed consent was not required for the conduct of this program. All imaging data were fully deidentified, encrypted, and processed in compliance with Health Insurance Portability and Accountability Act (HIPAA)/Health Information Technology for Economic and Clinical Health (HITECH) Act regulations and IEC 62443 cybersecurity standards, with built-in safeguards provided by the ViTAA software to ensure confidentiality.

## Consent for publication

A waiver of consent was requested and granted for this project as obtaining informed consent for this study met the criteria specified in Alberta's Health Information Act (HIA) for being unreasonable, impractical, or not feasible (Alberta's HIA, division 3, section 50: https://www.alberta.ca/health-information-act). All health information used in this study is previously collected, existing standard-of-care clinical data. No new information specific to the study was required. As a result, this study was unlikely to adversely affect the welfare of participants.

## Funding

The authors disclosed receipt of the following financial support for the research, authorship, and/or publication of this study: ViTAA Medical Solutions Inc sponsored the study, which was supported by a research grant from the 10.13039/100020453W. L. Gore & Associates Research Grant Committee (grant no. 315346).

## Disclosures

The authors declared the following potential conflicts of interest with respect to the research, authorship, and/or publication of this study: Dr A.L., has a Scientific Advisory Board Consultant Agreement with ViTAA Medical Solutions Inc. D.B. serves as VP Clinical Operations at ViTAA Medical Solutions and owns shares of ViTAA.
