# Supplementary material for: A Multicenter Case Review Program on the impact of ViTAA Regional Aortic Weakness Maps on clinical decision-making in abdominal aortic aneurysm management
Source: J Vasc Surg Cases Innov Tech. 2026 Oct 7;13(1):102546. doi: 10.1016/j.jvscit.2026.102546 (PMC13769224; doi:10.1016/j.jvscit.2026.102546)
Supplement: Supplementary file 1 [file mmc1.docx]

**Supplemental Materials**

**Supplemental Document 1.** Electronic Case Report Form (eCRF).

*Please collect and enter the following information into the designated EDC system.*

| **CRF Identification** | |
| --- | --- |
| ***Date of data entry*** | *Specify the date* |
| ***Investigator ID*** | *Type here the value* |
| ***CRF ID*** | *Type here the value* |
| ***Site ID*** | *Type here the value* |
| ***Patient ID*** | *Type here the value* |

| A. ***CT Scan Data*** | | |
| --- | --- | --- |
| ***CT Image ID*** | *Type here the value* | |
| ***Type of Study*** | *☐ Baseline* | *☐ Follow-up* |
| ***Imaging Technique*** | *☐ Multiphase gated CT scan* | *☐ Static CT scan* |

| B. **AAA Measurements from Standard of Care (SOC) assessment (CT scan)** | |
| --- | --- |
| **Maximal AAA Diameter (mm)** Type here the value ^mm^ | **Neck length (mm)** Type here the value ^mm^ |
| **Slice number** (from which the Max. AAA Diameter was obtained) | Type here the value |
| **Image viewing software used** (example: TeraRecon) | Type here the value |

| C. **Projected treatment plan SOC * *Please append the De-Identified EVAR case plan.*** | | | | | | |
| --- | --- | --- | --- | --- | --- | --- |
|  | **☐ Baseline** | | **☐ Follow-up** | |  | |
| **Diagnostic Intervention** | | |  | | | |
| **Image Technique** | | ☐ CT scan | ☐ Ultrasound | ☐ MRI | | |
|  |  | ☐ Angiogram | ☐ X-Ray | ☐ Echocardiogram | | |
|  |  | ☐ Other | Specify “Other” | | | |
| **Laboratory tests** | | ☐ Hematology | ☐ Biochemical analysis |  | |  |
|  |  | ☐ Other | Specify “Other” | | | |
| **Medical Intervention** | | ☐ Cholesterol lowering drugs | | ☐ Beta Blockers | | |
|  | | ☐ Angiotensin II receptor blockers | | ☐ Calcium channel blockers | | |
|  | | ☐ Other Anti-hypertensive agents | | ☐ Insulin | | |
|  | | ☐ Oral antidiabetic agents | | ☐ Anticoagulants/Anti-aggregation agents | | |
|  | | ☐ Antibiotic agents | | ☐ Anti-inflammatory agents | | |
|  | | ☐ Other treatments | | Specify “Other” | | |
| **When would you suggest scheduling assessments?** | | | ☐ 6 months | ☐ 12 months | | ☐ 24 months |
|  |  |  | ☐ Other | Specify “Other” | | |
| **Type of Intervention** | | ☐ Surgical Intervention | | ☐ Endovascular Intervention | | |
|  |  | ☐ Other | Specify “Other” |  | |  |
| **Type of procedure *(please append the De-identified EVAR case plan)**** | | ☐ Open repair | ☐ EVAR | ☐ FEVAR | | ☐ BEVAR |
|  |  | ☐ PMEG | ☐ Other | Specify Other | |  |
| **When would you suggest scheduling intervention?** | | | ☐ 6 months | ☐ 12 months | | ☐ 24 months |
|  |  |  | ☐ Other | Specify “Other” | | |
| **When would you suggest scheduling a follow-up?** | | | ☐ 6 months | ☐ 12 months | | ☐ 24 months |
|  |  |  | ☐ Other | Specify “Other” | | |

| D. **Projected treatment plan based on ViTAA RAW Maps assessment** | | | | | | | | | |
| --- | --- | --- | --- | --- | --- | --- | --- | --- | --- |
| **The *“Projected Care Plan SOC” of section C for the current subject is transcribed below.* Based on the data provided by VITAA (RAW maps and patient report), indicate what changes, if any, you would make to the *“Projected Care Plan SOC”* from section C by checking the boxes below.** | | | | | | | | | |
|  | | | | | | | | | |
|  | **☐ Baseline** | | | **☐ Follow-up** | | |  | | |
| **Diagnostic Intervention** | | | |  | | | | | |
| **Image Technique** | | ☐ CT scan | | ☐ Ultrasound | | ☐ MRI | | | |
|  |  | ☐ Angiogram | | ☐ X-Ray | | ☐ Echocardiogram | | | |
|  |  | ☐ Other | | Specify “Other” | | | | | |
| **Laboratory tests** | | ☐ Hematology | | ☐ Biochemical analysis | |  | |  | |
|  |  | ☐ Other | | Specify “Other” | | | | | |
| **Medical Intervention** | | ☐ Cholesterol lowering drugs | | | | ☐ Beta Blockers | | | |
|  | | ☐ Angiotensin II receptor blockers | | | | ☐ Calcium channel blockers | | | |
|  | | ☐ Other Anti-hypertensive agents | | | | ☐ Insulin | | | |
|  | | ☐ Oral antidiabetic agents | | | | ☐ Anticoagulants/ Anti-aggregation agents | | | |
|  | | ☐ Antibiotic agents | | | | ☐ Anti-inflammatory agents | | | |
|  | | ☐ Other treatments | | | | Specify “Other” | | | |
| **When would you suggest scheduling assessments?** | | | | ☐ 6 months | | ☐ 12 months | | ☐ 24 months | |
|  |  |  |  | ☐ Other | | Specify “Other” | | | |
| **Type of Intervention** | | ☐ Surgical Intervention | | | | ☐ Endovascular Intervention | | | |
|  |  | ☐ Other | | Specify “Other” | |  | |  | |
| **Type of procedure** | | ☐ Open repair | | ☐ EVAR | | ☐ FEVAR | | | ☐ BEVAR |
|  |  | ☐ PMEG | | ☐ Other | | Specify “Other” | | |  |
| **When would you suggest scheduling intervention?** | | | | ☐ 6 months | | ☐ 12 months | | | ☐ 24 months |
|  |  |  |  | ☐ Other | | Specify “Other” | | | |
| **When would you suggest scheduling a follow-up?** | | | | ☐ 6 months | | ☐ 12 months | | ☐ 24 months | |
|  |  |  |  | ☐ Other | | Specify “Other” | | | |
| Considering the use of the ViTAA RAW Maps software, which aspect of the RAW maps has had a significant impact on your decision-making process? | | | **☐** RAW score value | | ☐ RAW score location | | | ☐ RAW score distribution | |
|  | | | ☐ Other | | Specify “Other” | | |  | |

| E. **Data Collection Status** | | | | | |
| --- | --- | --- | --- | --- | --- |
| **Data collection completed** | | ☐ Yes ☐ No | | |  |
| ***Person collecting information Initials*** Type here the value | | |  | | |
| **Date** | Specify the date | | |  |  |
| Comments | Type here the value | | |  |  |

**Confidentiality Statement:** As part of the educational objective of this project, ViTAA is including the actual **clinical outcome** of each of the 5 patients. To safeguard the integrity of the research project, this clinical outcome MUST be **confidential** and MUST NOT be shared with other investigators.

F. **Person collecting information Signature**

_______________________________

Signature

____________

Date

***End of CRF***

**Supplemental Document 2.** Feedback Questionnaire.

| **Directive Questionnaire** | | | |
| --- | --- | --- | --- |
| **No.** | **Question** | **Answer** | **Comments** |
| 1 | It was easy to navigate through the software | ☐No ☐Yes  ☐Other | Click or tap here to enter text. |
| 2 | Were you able to easily visualize the 3D geometry of the aorta? | ☐No ☐Yes  ☐Other | Click or tap here to enter text. |
| 3 | Could you visualize, manipulate, and understand the strain map, the RAW map, the growth map and interact with them using your mouse and keyboard? | ☐No ☐Yes  ☐Other | Click or tap here to enter text. |
| 4 | Were you able to identify an area with high strain and high RAW in the iliac arteries? | ☐No ☐Yes  ☐Other | Click or tap here to enter text. |
| 5 | Were you able to perform different aortic measurements such as local diameters, centerline lengths, volumes, and caliper from a patient using the software? | ☐No ☐Yes  ☐Other | Click or tap here to enter text. |
| 6 | Were you able to compare the maps from the current study to a previous study of the same patient and recognize the differences in RAW score and changes in strain from scan A to B? | ☐No ☐Yes  ☐Other | Click or tap here to enter text. |
| 7 | Were you able to distinguish from the map where diametric growth has increased in the aneurysm? | ☐No ☐Yes  ☐Other | Click or tap here to enter text. |
| 8 | Were the reports useful and clearly understandable? | ☐No ☐Yes  ☐Other | Click or tap here to enter text. |
| 9 | Will the maps shown in this product be valuable in your practice? | ☐No ☐Yes  ☐Other | Click or tap here to enter text. |
| 10 | Is the ability to make measurements valuable to you? | ☐No ☐Yes  ☐Other | Click or tap here to enter text. |

**Instructions**: Do not leave any questions or spaces blank. Enter “N/A” if there is no information.

| **Open-Ended Questions** | | |
| --- | --- | --- |
| No. | Question | Answer |
| 3 | What did you find difficult or challenging about the maps? | Click or tap here to enter text. |
| 4 | What value does the overall software bring to you and your practice? | Click or tap here to enter text. |
| 5 | Do you suggest any areas for improvement on the user interface? | Click or tap here to enter text. |

**Annex**

A. Relative increases in procedures following ViTAA assessment.

*Percentages of patients deemed to need procedures after SOC and ViTAA assessments.*

| Time | SOC | ViTAA | Relative Increase |
| --- | --- | --- | --- |
| Baseline | 16.2 | 27.0 | 66.7% |
| Follow-up | 22.7 | 42.7 | 88.2% |

B. Changes in procedures following ViTAA assessments at baseline.

*Changes in recommendations following ViTAA.*

| **SOC → ViTAA** | **n/N** | **Percent** |
| --- | --- | --- |
| Surgical → Endovascular | 1/74 | 1.4% |
| Surgical → No Procedure | 1/74 | 1.4% |
| Endovascular → Surgical | 1/74 | 1.4% |
| Endovascular → Endovascular | 8/74 | 10.8% |
| Endovascular → No Procedure | 1/74 | 1.4% |
| No Procedure → Surgical | 2/74 | 2.7% |
| No Procedure → Endovascular | 8/74 | 10.8% |
| No Procedure → No Procedure | 52/74 | 70.3% |

C. Changes in procedures following ViTAA assessments at follow-up.

*Changes in recommendations following ViTAA.*

| **SOC → ViTAA** | **n/N** | **Percent** |
| --- | --- | --- |
| Surgical → Surgical | 2/75 | 2.7% |
| Surgical → No Procedure | 1/75 | 1.3% |
| Endovascular → Surgical | 2/75 | 2.7% |
| Endovascular → Endovascular | 11/75 | 14.7% |
| Endovascular → No Procedure | 1/75 | 1.3% |
| No Procedure → Surgical | 6/75 | 8.0% |
| No Procedure → Endovascular | 11/75 | 14.7% |
| No Procedure → No Procedure | 41/75 | 54.7% |

D. Percentages of assessment changes following presentation of ViTAA information.

*Proportion of Procedure Recommendation Changes when ViTAA was Provided.*

| **Timepoint** | **Percent** | **CI** |
| --- | --- | --- |
| Baseline | 18.9% | 10.0% – 35.8% |
| Follow-up | 28.0% | 19.1% – 41.0% |
