# Supplementary material for: A Multicenter Case Review Program on the impact of ViTAA Regional Aortic Weakness Maps on clinical decision-making in abdominal aortic aneurysm management
Source: J Vasc Surg Cases Innov Tech. 2026 Oct 7;13(1):102546. doi: 10.1016/j.jvscit.2026.102546 (PMC13769224; doi:10.1016/j.jvscit.2026.102546)
Supplement: Supplementary file 2 [file mmc2.docx]

**Supplemental Table I.** The percentage of physicians that responded “Yes” to each of the ten questions of the directive questionnaire section of the feedback form.

| feedback questions | percentage of physicians that responded yes |
| --- | --- |
| Q1. It was easy to navigate through the software. | 80 |
| Q2. Were you able to easily visualize the 3D geometry of the aorta? | 100 |
| Q3. Could you visualize, manipulate, and understand the strain map, the RAW map, the growth map and interact with them using your mouse and keyboard? | 100 |
| Q4. Were you able to identify an area with high strain and high RAW in the iliac arteries? | 73 |
| Q5. Were you able to perform different aortic measurements such as local diameters, centerline lengths, volumes, and caliper from a patient using the software? | 87 |
| Q6. Were you able to compare the maps from the current study to a previous study of the same patient and recognize the differences in RAW score and changes in strain from scan A to B? | 100 |
| Q7. Were you able to distinguish from the map where diametric growth has increased in the aneurysm? | 87 |
| Q8. Were the reports useful and clearly understandable? | 73 |
| Q9. Will the maps shown in this product be valuable in your practice? | 73 |
| Q.10 Is the ability to make measurements valuable to you? | 87 |

**Supplemental Table II.** List of participating physicians, their geographic locations, and assigned cases. Upon completion of the study and subsequent analysis, participants assigned to review the same patient cases were grouped into sub-teams, within which they collaboratively reached a consensus regarding each case. The hyphen indicates physicians who did not contribute to the final case consensus after completion of the Case Review Program.

| Participant | Geographic Location | Assigned case |
| --- | --- | --- |
| P1 | Boston, Massachusetts | CRP-002 |
| P2 | Dallas, Texas | CRP-001 |
| P3 | McLean, Virginia | CRP-003 |
| P4 | Norfolk, Virginia | CRP-001 |
| P5 | Dallas, Texas | CRP-001 |
| P6 | Nashville, Tennessee | _ |
| P7 | Philadelphia, Pennsylvania | _ |
| P8 | Houston, Texas | _ |
| P9 | Toronto, Ontario, Canada | _ |
| P10 | Jacksonville, Florida | CRP-003 |
| P11 | Rochester, Minnesota | CRP-004 |
| P12 | Rochester, Minnesota | CRP-005 |
| P13 | Rochester, New York | _ |
| P14 | Chapel Hill, North Carolina | CRP-005 |
| P15 | Los Angeles, California | CRP-004 |
